# Integrative Analysis of Histopathological Images and Genomic Data in Colon Adenocarcinoma

**DOI:** 10.3389/fonc.2021.636451

**Published:** 2021-09-27

**Authors:** Hui Li, Linyan Chen, Hao Zeng, Qimeng Liao, Jianrui Ji, Xuelei Ma

**Affiliations:** ^1^ Department of Biotherapy, State Key Laboratory of Biotherapy, Cancer Center, West China Hospital, Sichuan University, Chengdu, China; ^2^ West China Hospital, West China School of Medicine, Sichuan University, Chengdu, China

**Keywords:** colon adenocarcinoma, histopathological features, genomic data, random forest, prognosis

## Abstract

**Background:**

Colon adenocarcinoma (COAD) is one of the most common malignant tumors in the world. The histopathological features are crucial for the diagnosis, prognosis, and therapy of COAD.

**Methods:**

We downloaded 719 whole-slide histopathological images from TCIA, and 459 corresponding HTSeq-counts mRNA expression and clinical data were obtained from TCGA. Histopathological image features were extracted by CellProfiler. Prognostic image features were selected by the least absolute shrinkage and selection operator (LASSO) and support vector machine (SVM) algorithms. The co-expression gene module correlated with prognostic image features was identified by weighted gene co-expression network analysis (WGCNA). Random forest was employed to construct an integrative prognostic model and calculate the histopathological-genomic prognosis factor (HGPF).

**Results:**

There were five prognostic image features and one co-expression gene module involved in the model construction. The time-dependent receiver operating curve showed that the prognostic model had a significant prognostic value. Patients were divided into high-risk group and low-risk group based on the HGPF. Kaplan-Meier analysis indicated that the overall survival of the low-risk group was significantly better than the high-risk group.

**Conclusions:**

These results suggested that the histopathological image features had a certain ability to predict the survival of COAD patients. The integrative prognostic model based on the histopathological images and genomic features could further improve the prognosis prediction in COAD, which may assist the clinical decision in the future.

## Introduction

Colon adenocarcinoma (COAD) is the second most frequent malignancy in developed countries (1). In recent years, the incidence of COAD has been rising around the world. Although the survival rate of COAD has been improved greatly in the recent years, even in the European countries with the highest survival rate, the 5-year survival rate is no more than 60% (2). Currently, the most effective and recognized therapy of COAD is radical resection. Adjuvant treatment is designed to assist radical surgery, reduce the risk of recurrence, and improve the survival rates (3). Among the potential factors affecting the prognosis of COAD, the depth of tumor infiltration into the intestinal wall and the involvement of lymph nodes are the most important, which are also the basis of the clinical staging system (4). Therefore, accurate pathological diagnosis based on histopathological sections is critical for the prognostic prediction and therapy strategies of COAD.

Histopathological images contain a great deal of information about tumors, including the nature of the lesions, histological classification, and grade of malignancy. Therefore, histopathological diagnosis is often regarded as the gold standard, and has an irreplaceable status in clinical practice (5). Nevertheless, in many regions of the world, the number of pathologists and the services they are able to provide may not meet the needs of an adequate pathological diagnosis (6). The research and development of the digital whole slide imaging (WSI) system enable pathological sections to be read digitally, breaking the limitations of traditional microscopes. In addition, the application of computer aided diagnosis (CAD) based on the histopathological images promotes the intellectuality of pathological diagnosis, and thereby improves the diagnostic efficiency and accuracy (7). The computerized intelligent histopathologic image analysis system has been applied to breast (8), lung (9, 10), colon (11), and prostate (12) cancers due to its potential to identify novel tumor biomarkers.

The advantages of histopathological images in the prediction of tumor prognosis have been widely recognized. However, considering the complexity of molecular mechanisms affecting tumor prognosis, single-source predictors are far from adequate in prognostic modeling. Researchers have attempted to combine predictors from multiple sources to improve tumor prognostic models. For the past few years, the widespread application of high-throughput sequencing technology has promoted the research of serial analysis of gene expression, so that gene expression characteristics can be used for the prognosis prediction in cancers (13, 14). The information revealed by the cancer omics profile and histopathological images is not only relatively independent but also has commonality to a certain extent. The morphological features of tumor cells and histological structure of the tumor microenvironment can be influenced by molecular changes, individual immune function, and environment (15–17). For instance, a previous study (18) has found that there is a significant correlation between the TP53 mutation and pathological characteristics of tumor cells in lung adenocarcinoma. Another study demonstrated the correlation between the amplifications of PDGFRA, EGFR, MDM2, and specific image features in glioblastoma (19). Some researchers have combined radiomics and genomics to predict the clinical outcomes of cancers (20–22). For example, Schiano C. et al. integrated imaging parameters from hybrid 18F-FDG-PET/MRI with the expression level of Yin Yang 1 to predict early metastases of breast cancer (20). It is also feasible to combine the histopathological features with cancer omics to optimize the prognostic models. At present, the prognostic models based on the genomic data and histopathological image features have obtained a superior prediction performance in renal cell carcinoma (23), breast cancer (24), and other early-stage cancers (25), etc.

In this study, all of the whole-slide histopathological images were downloaded from The Cancer Imaging Archive (TCIA, http://www.cancerimagingarchive.net/) database and cropped into 1,000 x 1,000 pixel sub-images. TCIA collects, provides, and manages affluent cancer image data supported by 28 agencies, and can provide researchers with publicly available imaging data and unique imaging resources (26, 27). The mRNA expression profiles and clinical data of COAD patients were attained from The Cancer Genome Atlas (TCGA, https://portal.gdc.cancer.gov/) database. TCGA is one of the largest and richest publicly funded projects designed to build a comprehensive genetic map of the cancer genome (28). We extracted the histopathological image features through CellProfiler, an open-source modular image analysis software. CellProfiler can convert color image into grayscale and extract a number of features from identified cells or subcellular regions, including size, shape, intensity, and texture. We used the least absolute shrinkage and selection operator (LASSO) and support vector machine (SVM) models to identify the pathological features correlated with prognosis. Totally, five prognostic features were obtained by taking the intersection of the pathological features filtrated by the two algorithms. To further explore the potential correlation between the pathological and genomic features of COAD, we performed weighted gene co-expression network analysis (WGCNA) to identify the co-expression gene module that correlated most with the prognostic pathological image features. Finally, we utilized the random forest (RF) method to integrate the pathological features and genomic data to establish an integrative prognostic model and validated the model by the test set.

## Materials and Methods

### Data Source and Downloads

Totally, 719 whole slide histopathological images of 218 patients were downloaded from TCIA. The histopathological tissue slides were all formalin-fixed and paraffin-embedded to preserve the cell morphology as much as possible, ensuring that they were suitable for image feature recognition.

The mRNA expression data of HTSeq-count and clinical information of COAD patients were downloaded from TCGA. In total, we obtained 478 samples with mRNA sequencing data from TCGA, and 459 of them had clinical information. There were 19,754 genomic features in each sample (**Table S1**). Variance-stabilizing transformation (VST) was used to normalize the mRNA sequencing data with the R package DESeq2 for further analysis in WGCNA.

### Extraction of Histopathological Imaging Features

**Figure 1:**
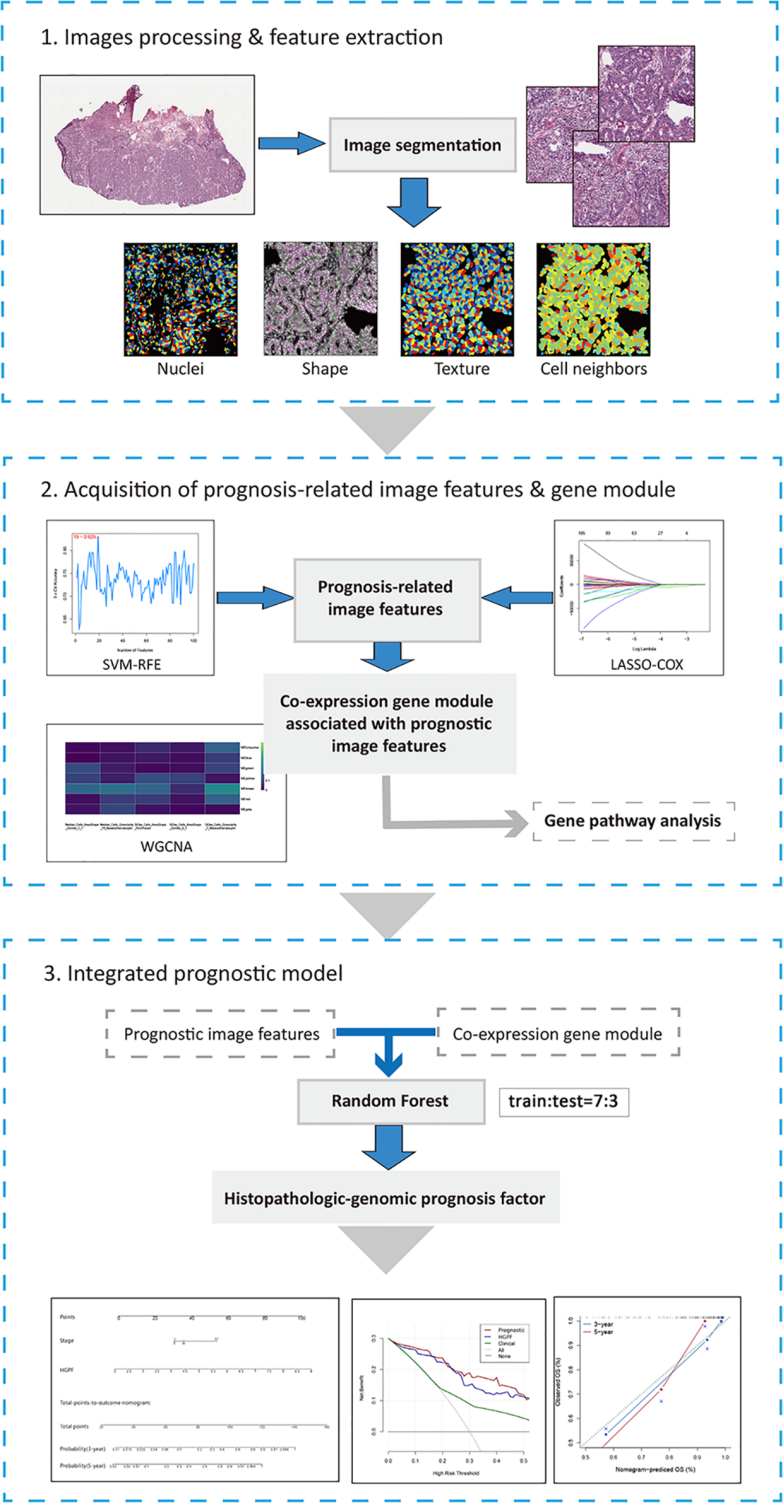
The workflow chart of key steps in this study. The whole-slide histopathological images of colon cancer were evenly cropped into sub-images of 1000×1000 pixels. After processing and selecting these sub-images, several image features were extracted by CellProfiler for further analysis. Then, LASSO-COX regression and SVM-RFE were performed to acquire prognosis-related image features. And WGCNA was used to identify co-expression gene modules associated with prognostic features. Histopathological features and genomic data were integrated into histopathological-genomic prognosis factor (HGPF) by random forest method with 10-fold cross-validation (the sample ratio of training set and test set is 7:3). Performance of the prediction model was evaluated.

The flowchart of processing histopathological images, extracting imaging features, and establishing an integrative prognostic model is shown in **Figure 1**.

In order to extract the image features from the whole slide histopathological images, the image processing procedure consisted of three steps. Firstly, since the size of each pathological image was too large to be used directly for feature extraction, we cropped each image evenly into 1,000 × 1,000 pixels sub-images and saved them in tiff image format using Openslide Python library (29). In this process, sub-images containing more than 50% white background were excluded. To eliminate the sample selection bias and reduce computing amount, we randomly selected 20 sub-images from the remaining sub-images for the next step. Cropping and random selection of images have been widely used in the processing of the whole slide images (9, 18, 24).

Secondly, we applied CellProfiler (30) to extract features from each sub-image. The hematoxylin-eosin staining makes the cell nuclei and cytoplasm appear different colors in the histopathological images. A total of 656 features were the output for each sub-image. These features were different from the well-known classical pathological characteristics such as cellular basophilic, eosinophilic, nuclear atypia, and mitotic counts, which cannot be recognized by visual inspection. After further removing irrelevant features such as file sizes and execution information, 590 features were used in the following workflow.

Thirdly, we calculated the average value of 590 features extracted from 20 sub-images for each slide. When a patient had more than one slide, the mean values over those slides were further calculated.

It should be emphasized that the purpose of our study was not to concretely explain the relationship between these image features and COAD, but to quest the optimal combination of features to establish an integrative prognostic model of COAD. Therefore, the lack of definite biological interpretations would not prevent us from conducting further reasonable analysis.

### Acquisition of Prognosis-Related Features

By using the R package “e1071” and “glmnet” on the R version 3.6.3 software, the support vector machines recursive feature elimination (SVM-RFE) and LASSO-Cox algorithms were employed to filtrate the prognostic image features most correlated with the prognosis of COAD. Here, we used 5-fold cross-validation in both the LASSO-Cox and SVM-RFE algorithms. LASSO constructs a penalty function, and compresses the insignificant variable coefficient to 0 to achieve the purpose of variable selection. By customizing the optimal value of the parameter lambda (λ), the user can control the balance between the sparsity (how many features are produced) and high prediction accuracy and minimum cross validation error. Image features with nonzero coefficients were finally regarded as the prognostic features and used to fit the regression model.

SVM-RFE is a backward feature selection machine learning method on the basis of SVM. In the training set, SVM-RFE ranked the pathological image features in a descending order of importance, iteratively eliminated the minimum features, and trained the model with the remaining features until all features were removed. When running the 5-fold cross-validation on SVM-RFE, feature selection was performed by defining the high-risk (survival time less than 12 months) and low-risk patients (survival time more than 60 months) as the training samples. In the SVM-RFE model, the maximal cross-validated accuracy was adopted as the evaluation index to confirm the optimal feature subset related to the prognosis. The optimal subset of features obtained by SVM-RFE was intersected with the results of LASSO regression to obtain the pathological features most relevant to the prognosis.

### Co-Expression Gene Module Analysis

WGCNA is an effective means to identify the co-expression gene modules by clustering the highly correlated genes, and perform correlation analysis between the modules and phenotypes to explore the potential marker genes of cancer (31). Based on the normalized mRNA profiles, WGCNA was employed to construct the co-expression gene network and explore the co-expressed gene module most correlated with the pathological prognostic features defined by machine learning algorithms. We calculated the interaction coefficient between genes and then computed the topological overlap measure (TOM) using the adjacency matrix. The co-expression network was constructed based on the W matrix to determine the co-expression gene modules. During this process, modules with statistical significance (p < 0.05) were regarded as prognosis-related modules. To further explore the interrelationship among the genes in the prognosis-related modules, we performed Gene Ontology (GO) enrichment analysis with Metascape (http://metascape.org). In this process, default thresholds were applied for pathway analysis.

### Establishment of an Integrative Prognostic Model

Based on the histopathological features and genomic data of COAD patients, we applied random forest algorithms with 1,000 decision trees by the R “randomForestSRC” package to construct an integrative prognostic model. RF is a classifier containing multiple decision trees and each tree is built on an independent bootstrap training set. The output category is determined by the mode of the output category of individual trees. RF has great advantages over other algorithms in high-dimensional data processing. It can process high-dimensional data without deleting variables, and can evaluate the predictive ability of each feature. Meanwhile, the unbiased estimation of the generalization error generated by internal cross validation ensures high accuracy. The randomness of training samples for each decision tree and the selection of variables for splitting at each node can reduce overfitting.

The samples were randomly divided into 10 parts, including 7 parts of the training set (n = 140) and 3 parts of the test set (n = 59). The ratio of 7:3 is commonly used in machine learning algorithms (32–34). The 10-fold cross-validation was used during model constructing. Based on the training set, we constructed the integrative prognostic model with the pathological image features and mRNA expression profiles, which was defined as the histopathological-genomic prognosis factor (HGPF) model. The test set was then used to validate the prediction performance of the HGPF model. Afterwards, time-dependent receiver operating characteristic (ROC) curve was plotted and the 1-, 3-, and 5-year area under curves (AUCs) were calculated according to the average accuracies of the 10-fold cross-validation. We then used the RF model to estimate the survival risk of each patient and obtain the risk score of each patient. Based on the median of the risk score, the training set and test set can be divided into the high-risk score group and low-risk score group, respectively. Kaplan-Meier analysis and the log-rank test was used to compare the survival difference between the two groups.

After univariate Cox regression, we incorporated meaningful results (p < 0.05) into the multivariate Cox regression analysis. Two predictive factors, HGPF and tumor stage of patients, were used in the development of the prognostic nomogram. In the nomogram, scores were assigned to the predictive factors according to the impact of the predictors on the survival outcome (the value of regression coefficient). Finally, the total score of each patient was associated with the survival probability through the function conversion.

## Result

### Patient Characteristics

A total of 199 COAD patients (112 male and 87 female) were included in this study. Histopathological images, mRNA expression data, and clinical information were downloaded from TCIA and TCGA. The median age of patients at first diagnosis was 71.0 years old (range 36–89 years). There were 167 patients who survived and 32 patients who died at the last follow-up. The median survival time was 24.5 months. Patient characteristics are shown in **Table 1** and detailed clinical information of patients are shown in **Table S2**.

**Table 1:** Demographic and clinical characteristics of patients.

Characteristic	Total (n = 199)	Train (n = 140)	Test (n = 59)	P-value
Age: median (range)	71.0 (36–89)	72.0 (36–89)	68.0 (41–86)	0.599
Gender				
Male	112 (56.3%)	78 (55.7%)	34 (57.6%)	
Female	87 (43.7%)	62 (44.3%)	25 (42.4%)	0.876
T classification				
T1–T2	39 (19.6%)	25 (17.9%)	14 (23.7%)	
T3–T4	160 (80.4%)	115 (82.1%)	45 (76.3%)	0.336
N classification				
N0	124 (62.3%)	84 (60.0%)	40 (67.8%)	
N1–N2	75 (37.7%)	56 (40.0%)	19 (32.2%)	0.339
M classification				
M0	151 (75.9%)	105 (75.0%)	46 (78.0%)	
M1	29 (14.6%)	20 (14.3%)	9 (15.3%)	
Mx	15 (7.5%)	12 (8.6%)	3 (5.1%)	0.690
NA	4 (2%)	3 (2.1%)	1 (1.7%)	
TNM stage				
I–II	115 (57.8%)	78 (55.7%)	37 (62.7%)	
III–IV	78 (39.2%)	57 (40.7%)	21 (35.6%)	0.523
NA	6 (3.0%)	5 (3.6%)	1 (1.7%)	
OS(d): median	735.0	737.5	731.0	0.448
Event				
Alive	167 (83.9%)	114 (81.4%)	53 (89.8%)	0.204
Dead	32 (16.1%)	26 (18.6%)	6 (10.2%)	

### Acquisition of Histopathological Images Features

CellProfiler transforms color images into grayscale images and measures image features from 10 aspects, including the correlation between intensities in different images, image area occupied, image granularity, image intensity, image quality, object intensity, object neighbors, object radial distribution, object size, shape, and texture. Texture reflects the degree and nature of the image or object textures through measuring the intensity variations in grayscale images. Image granularity is a texture measurement that outputs the spectra of the fitting degree between the size measures of the structure elements and image texture. Object size shape measures several area and shape features of each identified object in the image, such as area, perimeter, formfactor, solidity, Euler’s number, and orientation. For example, form factor measures the object shape with the formula “4*π*Area/Perimeter2”. Zernike shape features contain a series of 30 shape features based on Zernike polynomials from order 0 to order 9.

Finally, we extracted 590 image features from each sub-image and calculated the average value of representative sub-images for each corresponding slide.

### Prognosis-Related Features Identification and Co-Expression Gene Module Selection

**Figure 2:**
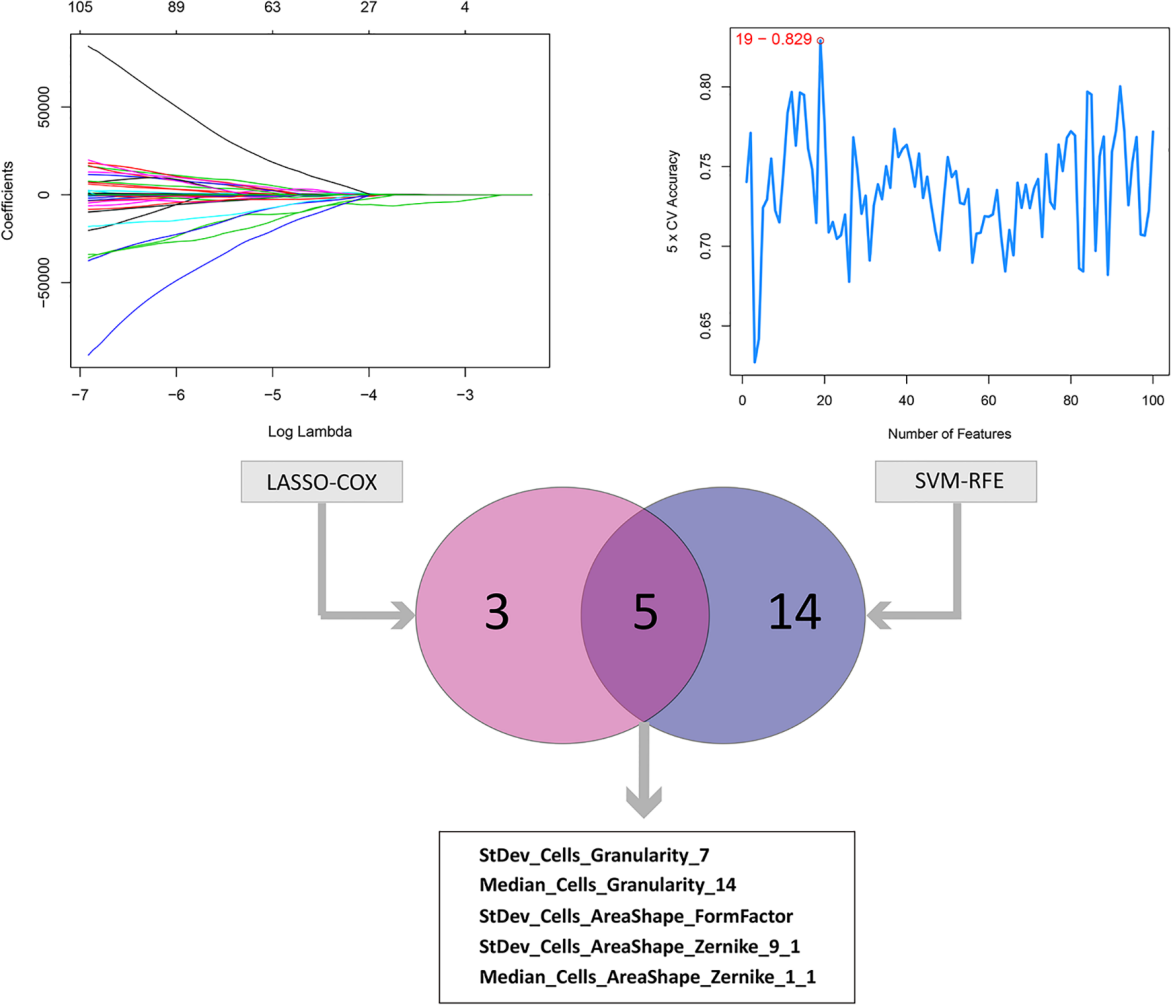
LASSO and SVM-RFE algorithms for the identification of prognosis-related features. The LASSO-COX regression screened out 8 prognostic features while the SVM-RFE algorithm identified 19 features at the point highlighted, which indicated the maximal cross-validated accuracy. Taking the intersection of the two result to obtain the 5 prognosis-related features.

**Figure 3:**
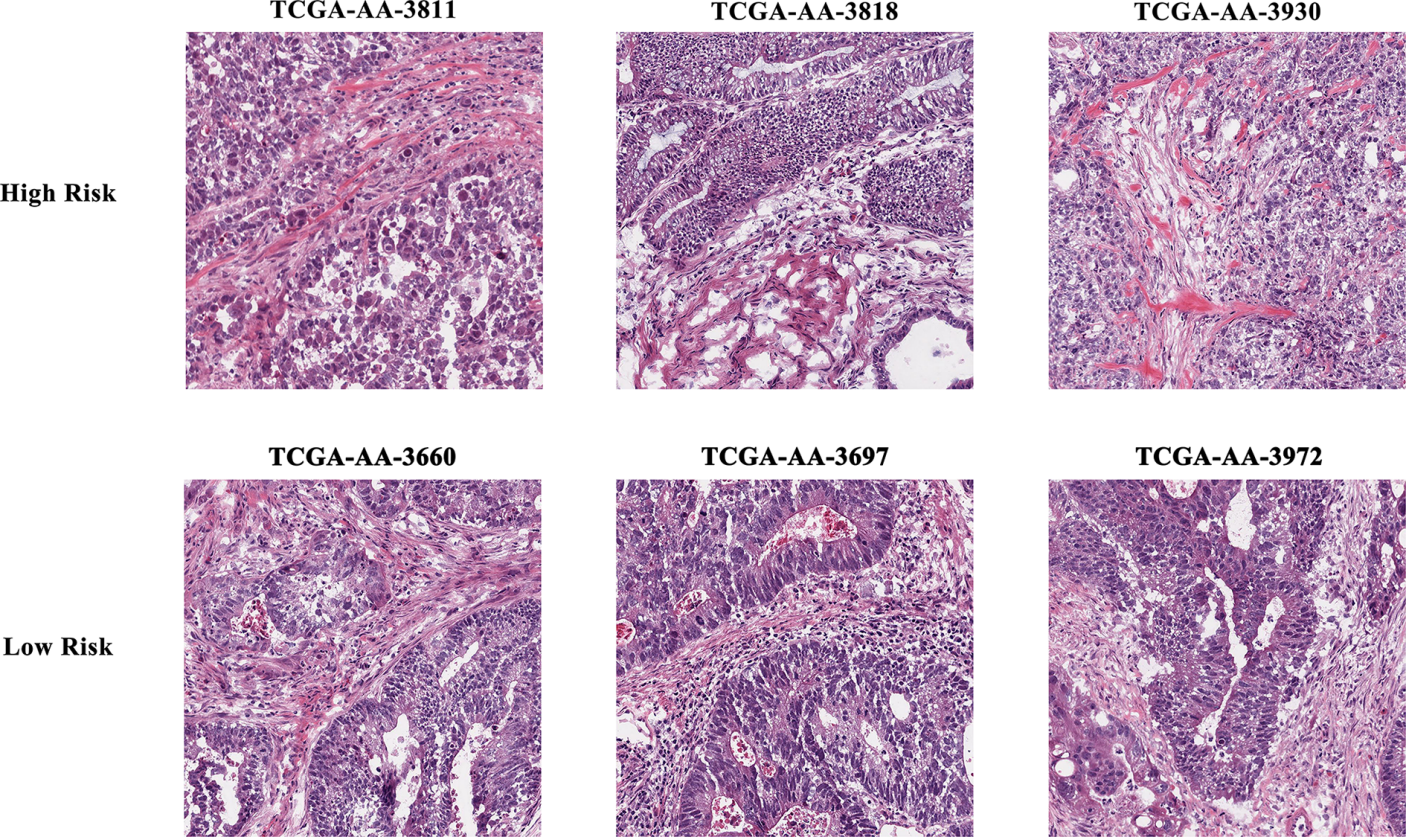
Example of selected histopathological sub-images in both high- and low-risk groups.

**Figure 4:**
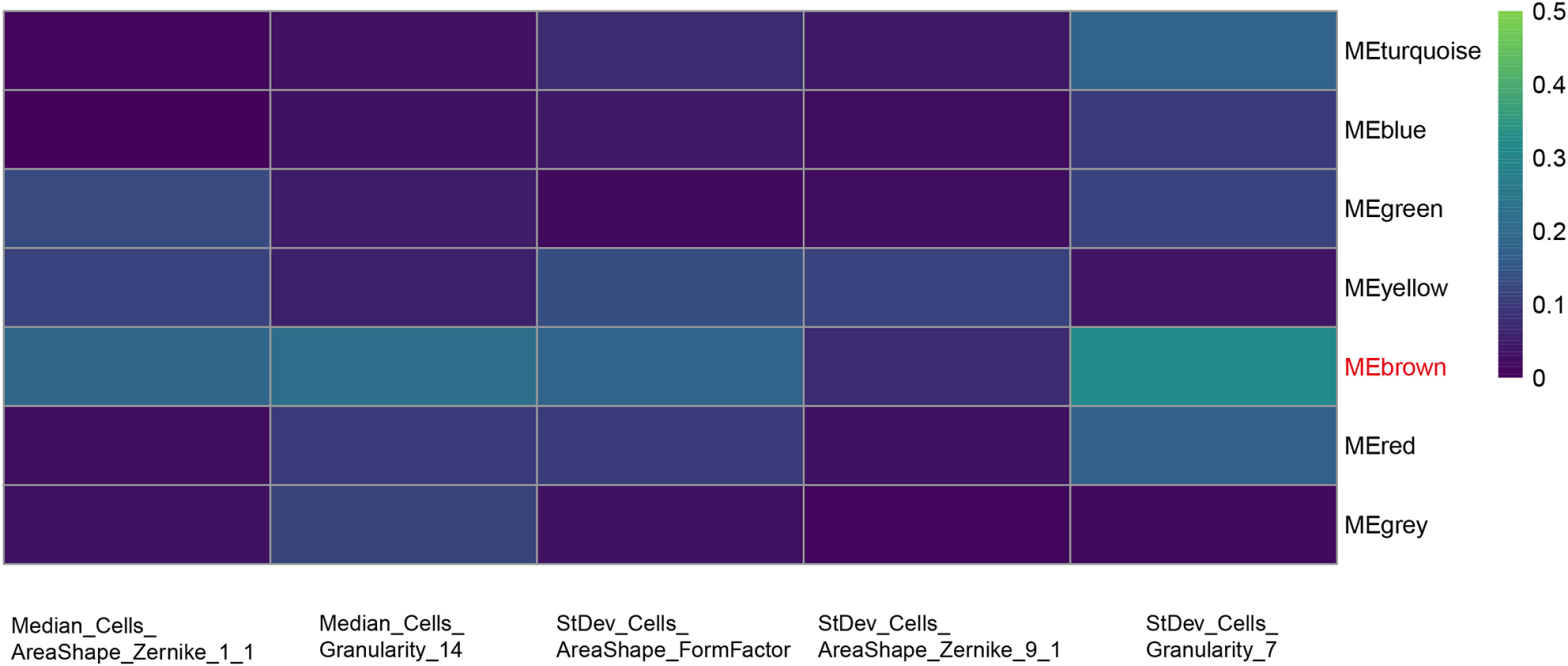
Selection of the co-expression gene module related with the prognostic features. Heat map of the relationship between the five prognosis-related features and co-expression gene modules by WGCNA. Brown module shows the most outstanding correlation with the imaging features.

The results of data dimension reduction through LASSO-Cox and SVM-RFE are shown in **Figure 2**. The optimal subset of features determined by the maximal cross-validated accuracy contained 19 features after feature elimination by the SVM-RFE algorithm. The LASSO-Cox regression identified eight prognostic features. We then intersected the results of the two algorithms to obtain five features (two Zernike shape features, two Granularity features, and one formfactor feature), which were defined as the prognostic image features of COAD. The examples of selected histopathological sub-images in both high-risk and low-risk groups are presented in **Figure 3**. To identify the co-expression gene modules with prognostic significance, WGCNA was applied to evaluate the relationship between the five prognostic image features and co-expression gene modules. The strength of association was represented by different colors (**Figure 4**). Obviously, the brown module containing 372 genes had the most outstanding association with the image features. Therefore, the brown module was selected as the key module with prognostic significance to build the integrative prognostic model.

### Enrichment Analysis of the Key Gene Module

**Figure 5:**
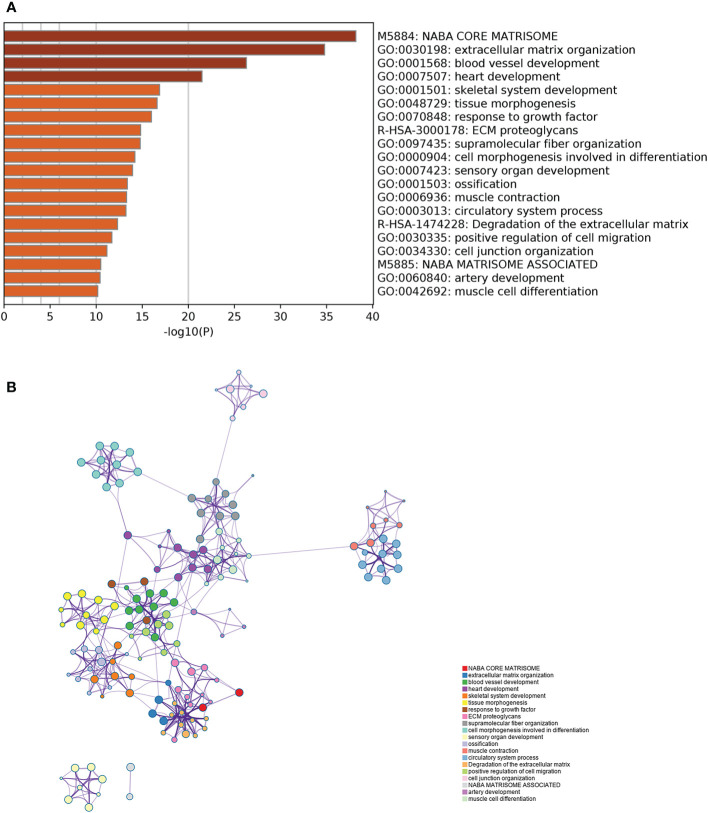
Gene Ontology enrichment analysis of the key gene module. **(A)** The top 20 GO terms which were significantly enriched. **(B)** The interrelationship and intrarelationship among the cluster of enriched terms. Each dot represents one term, and the color annotates its cluster identity.

**Figure 5A** lists the top 20 GO terms that were significantly enriched. The interrelationship among the 372 genes and their respective pathways is shown in **Figure 5B**. The results indicated that there were significant intrinsic associations among the biological function of these genes. In addition, most of them were enriched in biological processes such as blood vessel development, heart development, skeletal system development, and tissue morphogenesis. Several cellular components were also related, such as extracellular matrix (ECM) organization, ECM proteoglycans, and supramolecular fiber organization. Detailed enrichment results of 372 genes are shown in **Table S3**.

### Construction and Validation of the Integrative Prognostic Model

**Figure 6:**
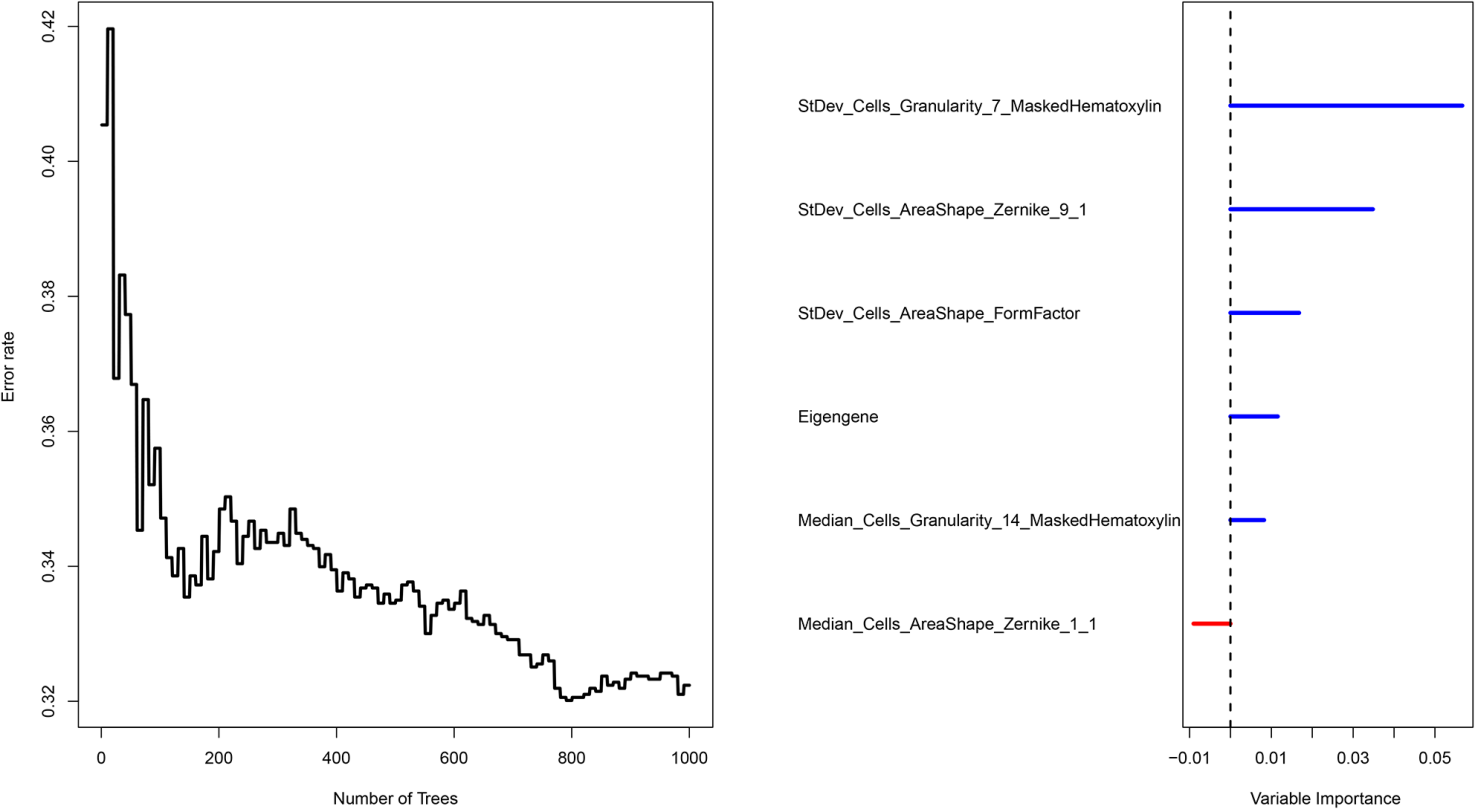
Survival prediction of the integrated model with the prognosis-related features and genomic data using random forest.

**Figure 7:**
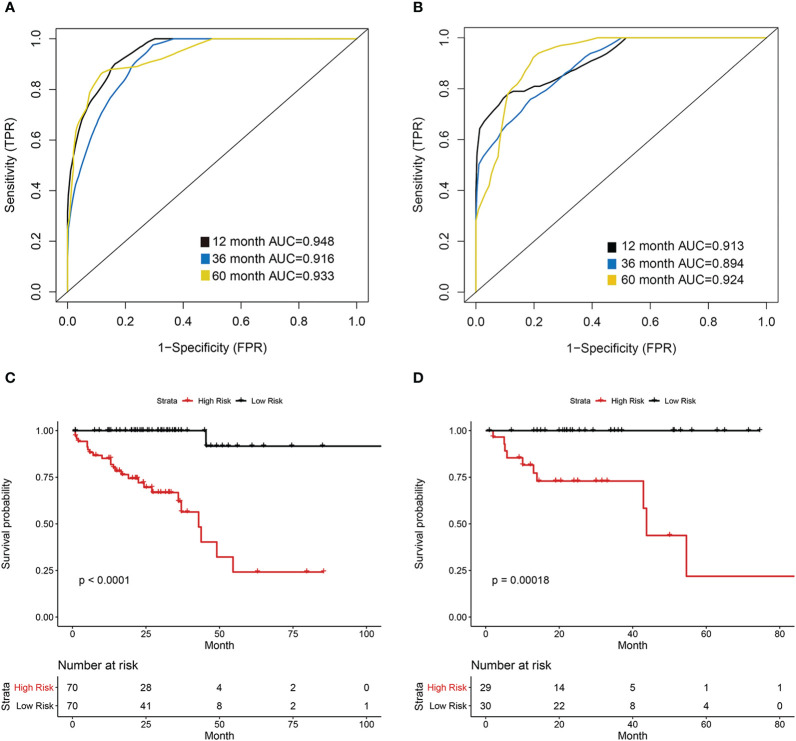
Prognostic model of the histopathological-genomic prognosis factor (HGPF). **(A)** The training group 1-, 3-, and 5-year area under the curve (AUC) of a time-dependent receiver operating curve (ROC). **(B)** The test group 1-, 3-, and 5-year area under the curve (AUC) of a time-dependent receiver operating curve (ROC). **(C)** Survival analysis of the training cohort separated into high- and low-risk groups. **(D)** Survival analysis of the test cohort separated into high- and low-risk groups.

The COAD patients were randomly divided into the training set (n = 140) and test set (n = 59). We next established a prognostic model using the prognostic image features and gene module in the training set, and validated its predictive value in the test set. The development of a random forest model and the importance of six variables are shown in **Figure 6**. Since the survival results include both the survival state and survival time, the time-dependent ROC curve can more comprehensively describe the predictive ability of the model over time. In the training set, the 1-, 3-, and 5-year AUCs were 0.948, 0.916, and 0.933 respectively (**Figure 7A**). In the test set, the 1-, 3-, and 5-year AUCs were 0.913, 0.894, and 0.924, respectively (**Figure 7B**). The predictive accuracy of the test set remained at a high level. Whereafter, patients were divided into the high-risk group and low-risk group by the median value of HGPF. The detailed results of random forest for the training set and test set are shown in the **Supplementary Material** (**Tables S4, S5**). The results of the Kaplan-Meier analysis demonstrated that the overall survival rate of low-risk score patients was significantly better than that of high-risk score patients in both the training set (p < 0.0001, **Figure 7C**) and test set (p = 0.00018, **Figure 7D**).

**Figure 8:**
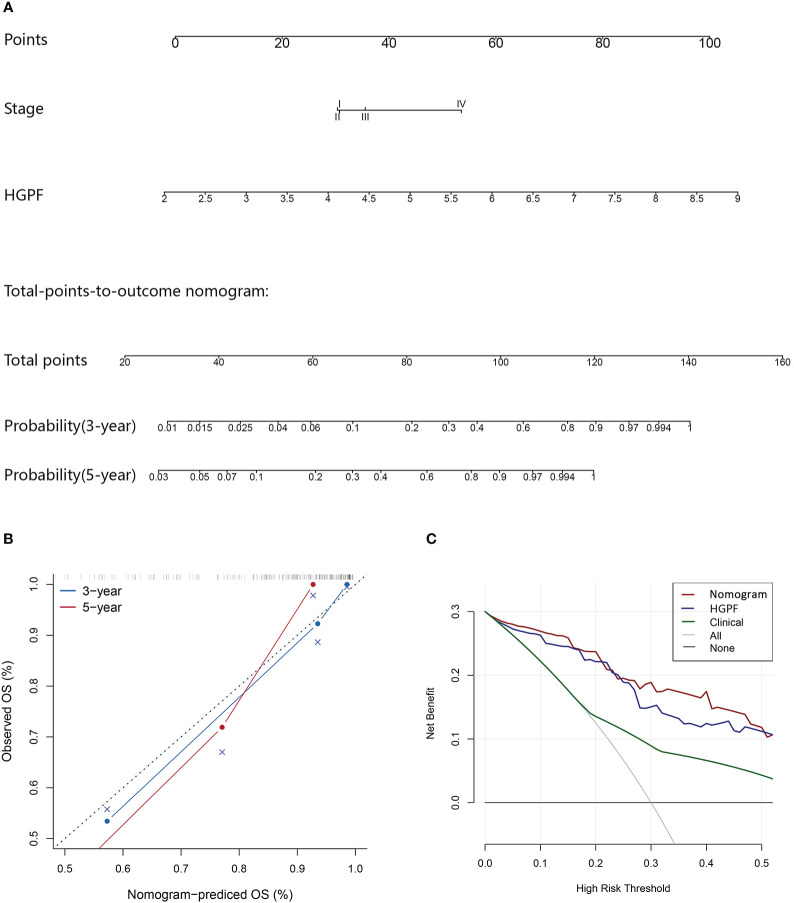
Evaluation of the predictive performance of the integrated prognostic model. **(A)** The prognostic nomogram incorporates the histopathological-genomic prognosis factor (HGPF) and tumor stage of patients. **(B)** Assessment of nomogram prediction accuracy. **(C)** Decision curve analysis (DCA) shows the predictive effects of different models. The integrated model has a better prediction ability than others.

A nomogram scoring system incorporating the HGPF and tumor stage of patients was constructed using the Cox regression model (**Figure 8A**). Patients were scored according to the weights of the two predictors, and the 3- and 5-year overall survival probabilities were predicted. The calibration curve demonstrated that the nomogram had a high fitting degree for the prediction of the 3- and 5-year overall survival compared to the actual outcomes (**Figure 8B**). Moreover, decision curve analysis (DCA) was used to evaluate the clinical benefit of each model, including the integrative prognostic model (HGPF risk score combined with tumor stage), HGPF model, and clinical model. The integrated model had a better net benefit than others in the DCA analysis (**Figure 8C**).

## Discussion

In this study, we extracted image features from whole-slide histopathological images by CellProfiler and identified five prognostic image features with machine learning algorithms. We also identified a prognosis-related module by establishing a gene co-expression network. We detected significant intrinsic associations among the biological function of the genes in a prognosis-related module through enrichment analysis. Furthermore, the prognostic image features, co-expression gene module, and clinical information were integrated to construct a prognosis prediction model, which had a better prediction performance than other models. In summary, it is suggested that histopathological image features have a certain ability to predict patient survival, and multi-omics combination could further improve the prognosis prediction in COAD.

Our study identified five image features associated with the prognosis of COAD patients, including two Zernike shape features of the nuclei, two Granularity features, and a formfactor feature. It can be inferred that the differences in the texture and morphology of the pathological images may influence the prognosis of COAD. In addition to prognosis prediction, the discrepancies in the cell structure revealed by these image features may lead to the differences in the invasion activity of tumor cells. In bladder cancer, a staging diagnostic model based on tumor invasiveness were developed with the histopathological image features (35). This approach can also be applied to the accurate grading of other cancers (36). It was difficult for pathologists to distinguish these image features by the naked eye. Therefore, the application of computer algorithms to identify the histopathological features related to prognosis could reveal more underlying biological mechanisms of tumor development and progression in COAD.

After defining the prognostic image features, we constructed a gene co-expression network to identify the prognosis-related gene module and performed enrichment analysis to further explore the potential molecular pathways and mechanisms of genes in the brown module. Among the enriched signaling pathways, pathways associated with the tumor microenvironment were dominated such as ECM organization and blood vessel development. ECM is a key regulatory factor in the initiation of the TGF-β signaling pathway, and can determine the outcomes of cytokine action, such as inducing the epithelial-mesenchymal transition (EMT) (37, 38). EMT was regarded as a pivotal step for cancer cells to acquire the ability of migration and invasion (39, 40). Moreover, the changes of ECM organization may play a crucial role in tumor recurrence and therapeutic resistance (41, 42). The rapid proliferation of cancer cells leads to the formation of hypoxic areas in the tumor center. The tumors facilitate angiogenesis for further growth, which, in return, increases the need for new blood vessels (43). Epithelial cells with EMT will lead to the reduction of cell junctions, recombination of the cytoskeleton structure, and changes in the cell polarity and cell shape, which may lead to characteristic changes in the histopathological images (44). Considering the correlation between the five prognosis-related features and co-expression gene module, these enriched signaling pathways may be potential biological mechanisms correlated with the prognosis-related histopathological image features.

Our research further established a robust prognostic model using prognostic image features, prognosis related co-expression gene module, and clinical characteristics of COAD patients. Many previous studies have conducted extensive investigation and modeling using single omics, such as genomic signatures of COAD (45–47). In this study, we integrated the pathological image features and genomics of COAD for the first time and improved the prediction performance of single-source prognostic models. This method of combining the pathological images with genomics to predict survival has been applied in other tumors (23, 48). Some studies also found that integrative models could improve the prediction performance of genomics and other images modalities, such as magnetic resonance imaging and computerized tomography (49–51). However, on account of the changes in tumor molecular mechanisms are often reflected in cell morphology, pathological images may have a better insight, interpretability, and sensitivity than radiomic images.

To our best knowledge, this was the first time that histopathological images and genomics were integrated to predict the prognosis of COAD patients. Our research exploited a new feasibility for establishing prognostic models of COAD with multi-omics data, and conducted more utilization and excavation of histopathological image information. In addition, the analysis of signaling pathways may put forward a new direction for the potential biological mechanism of pathological morphological changes, which may provide a reference for the clinical prognosis and treatment strategies in COAD. However, this study still had some limitations and required further investigation. Although a significant prognostic value of the integrative model has been demonstrated in our validation, its accuracy and practicability still need to be verified by multi-center and large-scale studies. Secondly, the specific molecular mechanisms of the connection between the enriched signaling pathway and the prognostic model are still unclear and need further study.

## Conclusion

In conclusion, our study constructed a robust integrative model based on multi-omics features to predict the survival outcomes of patients with colon adenocarcinoma. This model deepened the cognition about the histopathological image information and may contribute to the clinical decision and treatment of colon adenocarcinoma. Moreover, the potential biological mechanisms of the histopathological image features affecting the survival outcomes need further exploration.

## Data Availability Statement

The original contributions presented in the study are included in the article/**Supplementary Material**. Further inquiries can be directed to the corresponding author.

## Author Contributions

HZ and HL are responsible for the conception and design of the research. LC is responsible for data downloading and sorting. HZ conducted the data processing. HL is responsible for editing the article and formatting. QL is in charge of the interpretation of data. JJ is in charge of submission and manuscript revision. All authors contributed to the article and approved the submitted version.

## Conflict of Interest

The authors declare that the research was conducted in the absence of any commercial or financial relationships that could be construed as a potential conflict of interest.

## Publisher’s Note

All claims expressed in this article are solely those of the authors and do not necessarily represent those of their affiliated organizations, or those of the publisher, the editors and the reviewers. Any product that may be evaluated in this article, or claim that may be made by its manufacturer, is not guaranteed or endorsed by the publisher.
